# Mendelian randomization study reveals the relationship between dietary factors and respiratory diseases

**DOI:** 10.1038/s41598-023-50055-x

**Published:** 2023-12-18

**Authors:** Wei Lai, Guorui Li, Dunyu Peng, Ning Li, Wei Wang

**Affiliations:** 1 Department of Thoracic Surgery, Renmin Hospital of Wuhan University, Wuhan, 430060 China; 2 Department of Anesthesiology, Renmin Hospital of Wuhan University, Wuhan, 430060 China

**Keywords:** Endocrinology, Health care, Risk factors

## Abstract

The existence of causal relationship between dietary factors and respiratory diseases is uncertain. We comprehensively investigated the association between dietary factors and respiratory diseases by using Mendelian randomization (MR). Genetic variants linked to dietary factors were selected as instrumental variables with genome-wide significance. These instrumental variables were obtained from large GWAS databases. These databases include Biobank, the FinnGen study, and other large consortia. We used multivariate MR analyses to control the effects of smoking and education. Median analysis was conducted to evaluate whether body mass index (BMI) played a role in dietary factors in respiratory diseases. Dried fruit intake was found to be associated with a decreased risk of chronic obstructive pulmonary disease (COPD) (OR: 0.211; 95% CI 0.117–0.378;* P* < 0.001) and asthma (OR: 0.539; 95% CI 0.357–0.815; *P* = 0.003). Conversely, pork intake was associated with an increased risk of idiopathic pulmonary fibrosis (IPF) (OR: 1.051*10^2^, 95% CI 4.354–2.56*10^3^, *P* = 0.004). However, no significant associations were observed between the 20 dietary factors and obstructive sleep apnea (OSA). In addition, multivariate MR analyses showed that the above results were unchanged in smoking and nonsmoking populations, while the effect of dried fruit intake on asthma was significantly attenuated after corrective education. The results of the mediator variable analysis indicated that BMI could serve as a mediator of the above results. This study found that dried fruits slowed the progression of COPD and asthma, while pork promoted IPF. However, no effect of dietary factors on OSA was found. Meanwhile, we showed that the above results were unchanged in smoking and non-smoking populations. In contrast, education could influence the role of diet on asthma, and BMI could be used as a mediating variable to influence the above results.

## Introduction

Respiratory diseases are one of the world's healthcare burdens and vast amounts of money are invested in research into respiratory diseases every year^1^. In developing countries, patients afflicted with respiratory diseases face economic challenges and an elevated risk of poor prognosis due to limited access to appropriate treatments. A growing body of observational studies has suggested that dietary factors may play a pivotal role in developing respiratory diseases^2,3^. Numerous cross-sectional and longitudinal studies consistently affirm that a rich intake of fruits, raw vegetables, and dietary fibers is inversely correlated with the risk of developing chronic obstructive pulmonary disease (COPD)^4–6^. In contrast, high consumption of processed meat increases the risk of COPD by 40% when compared to a lower consumption^7^. Similarly, previous studies have likewise found that alcohol intake, fruit intake, and fish intake are associated with asthma or asthma symptoms^8^. Different dietary habits may also play an important role in the development of obstructive sleep apnea (OSA). For example, the ketogenic diet (a food rich in fat, low in carbohydrates, and adequate in protein) is considered an alternative treatment for OSA in addition to medication with lifestyle changes^9^. However, most of these causal relationships come from observational studies, which are heavily confounded by reverse causation and confounding factors, and thus the results are not entirely reliable. Reverse causation may exist for some respiratory disorders that cause discomfort, which alcoholic patients may attempt to relieve by increasing alcohol dose or by ingesting more nicotine, despite the fact that it may not be a problem in studies for respiratory diseases^10^. Consequently, establishing a causal relationship between dietary and respiratory system diseases is crucial. In clinical practice, nutritional assessment and guidance, as well as nutritional interventions specific to the disease, are important means of managing respiratory system diseases. In addition, exploring the impact of dietary factors on respiratory system diseases can also promote the application of relevant non-pharmaceutical therapies.

As randomized clinical trial (RCT) studies are labor and financial-intensive and heavily disturbed by confounding factors, alternative methods of enhancing causal inference may help guide whether these trials are reasonable^11^. Mendelian randomization (MR) is a study design that follows Mendel’s laws of inheritance and uses single nucleotide polymorphisms (SNPs) as instrumental variables to infer the relationship between exposure and outcome^12^. Since genetic variation is randomly assigned at the time of gamete formation and conception according to Mendel’s laws, genetic variation can be used as a tool to randomly classify the study population into groups with higher or lower levels of exposure. MR has the advantage of using genes as instrumental variables. For example, the genetic effects of genotypes are relatively stable and not susceptible to environmental influences, and all genetic variation is determined during pregnancy, which occurs before onset of disease. Thus, MR can overcome the limitations of traditional observational studies that are susceptible to confounding and reverse causation^13^. Although MR designs have been used in recent years to investigate the relationship between dietary factors and lung cancer risk, causality remains unclear for a wide range of respiratory diseases such as COPD, idiopathic pulmonary fibrosis (IPF), asthma and OSA^14^. A comprehensive investigation of the impact of dietary factors on respiratory diseases is essential for developing non-pharmacological interventions in this area.

In this study, we first analyzed the effects of 20 dietary factors on COPD, IPF, asthma and OSA using two-sample MR . Due to the significant impact of smoking on respiratory diseases, we used multivariate MR analyses to explore the impact of these results in both smoking and non-smoking populations^15,16^. In addition, we controlled for education as it is a confounder of many current diseases^17^. Different dietary habits have different effects on body mass index (BMI), which can exist as a mediating variable in cardiovascular disease, so we equally explored whether BMI could act as a mediating variable^18^.

## Materials and methods

### Study design

**Figure 1:**
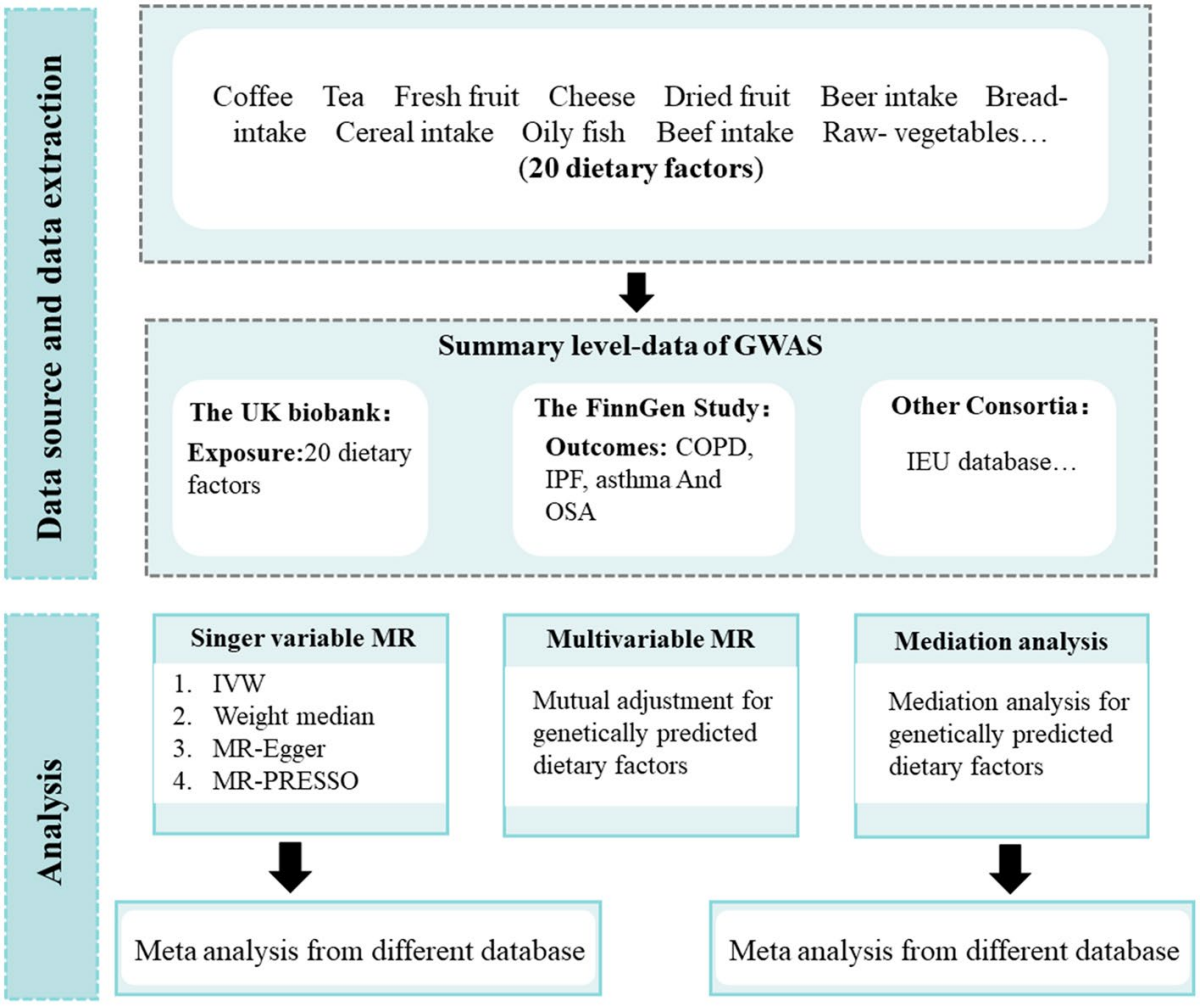
Overview of the study design.

Figure 1 presents an overview of our study design. We used two-sample MR analyses to examine the effects of 20 dietary factors on COPD, IPF, asthma and OSA. In addition, we utilized multivariate MR analyses to correct for the confounding factors of smoking and education. We equally explored whether BMI might act as a mediating variable. MR analysis utilizes genetic variants as instrumental variables, enabling us to estimate the causal association between dietary exposures and respiratory diseases^19^. To meet the assumptions of MR, the selected genetic variants must adhere to three critical criteria: (1): reliably and robustly related to the exposure, (2): independent of risk factor-outcome confounders, (3): influence the outcome only via exposure, and (4) influence the outcome only via the exposure^20^. The data for our analysis were derived from recently available genome-wide association studies (GWASs) and involved summary-level data.

## Data sources and SNPs selection

### Exposure data

We utilized summary-level data from a comprehensive collection of genetic consortia and the Pan UK Biobank (as of July 1, 2023) to investigate 20 exposures related to respiratory diseases. These exposures encompass a wide range of dietary factors, including coffee intake, tea intake, fresh intake, cheese intake, dried fruit intake, beer intake, bread intake, cereal intake, oily fish intake, beef intake, raw vegetable intake, saturated fatty acids, polyunsaturated fatty acids, lamb intake, pork intake, bacon intake, processed meat, red wine, non-oily fish intake, alcohol intake frequency, and cooked vegetable intake. We consider these dietary components as representative of global dietary habits, thus providing comprehensive coverage of diets worldwide. Supplementary Table S1 contains further detailed information regarding the exposure datasets.

### Outcome data

We obtained summary-level data on prevalent respiratory diseases, namely COPD, IPF, asthma, andOSA , from the FinnGen biobank. Supplementary Table S1 provides a comprehensive description of the data sources for the respective outcome datasets.

### Potential pleiotropy and potential mediators

We collected summary statistics data on smoking (sample = 607,291) and education (sample = 461,460) from separate datasets obtained from the IEU (Integrative Epidemiology Unit) database and the UK Biobank. This approach was implemented to avoid any population overlap between the exposures and outcomes datasets. Additionally, we acquired summary statistics from genome-wide association studies of potential mediators, specifically BMI and blood glucose . Detailed information on the data sources for both exposure and outcome datasets are available in Supplementary Tables S1, S7 and S9.

### SNP selection

In this study, we identified SNPs that were strongly associated with exposures and outcomes, with genome-wide significance (P < 5 * 10^–8^). These selected SNPs were situated in diverse gene regions and exhibited no significant linkage disequilibrium (r^2^ < 0.001), as determined using data from the 1000 Genomes European reference panel^21^. To assess potential confounding effects, we thoroughly searched for included instrumental variables using PhenoScanner^22,23^. Our MR analysis adhered to the necessary assumptions, and the outcome demonstrated genome-wide significance (P < 5 * 10^–8^). Supplementary Table S2 contains additional detailed information relevant to the instrumental variables used in our study.

### Strengthen instrumental variables

In order to minimize the impact of weak IVs on the causal analysis, we calculated the F-statistic using the following equation: F = R^2^ / (1 − R^2^) * (N − k − 1)/k^24^. Here, R^2^ means the proportion of risk factor variance explained by genotype, N denotes the sample size, and k is the number of instrumental variables used in the analysis. The calculation of R^2^ was performed using the formula: R^2^ = [beta. exposure^2^]/[se. exposure^2^ * N + beta. exposure^2^]^25^. An F-statistic value greater than 10 indicates a low probability of weak instrument bias, which implies that the instrumental variables have sufficient strength to generate reliable and unbiased causal estimates in MR analysis. The evaluation of weak instrument bias helps ensure the validity and robustness of our findings related to the causal relationships between the exposure (dietary factors) and the outcomes (respiratory diseases).

## Statistical analysis

### MR estimates using univariable MR

The main analyses were conducted using a random effects inverse variance weighted (IVW) approach, which provides precise causal estimates assuming that all variables are valid instrumental variables^26^. This choice was made based on its suitability for integrating effect estimates from multiple independent studies, effectively harnessing information from each study, and maintaining statistical validity. We used three statistical methods for sensitivity analyses, MR-Egger, weighted median, and MR-PRESSON^27–29^. Weighted median allows 50% of instrumental variables to violate MR assumptions in the presence of horizontal pleiotropy^30^. The MR-Egger intercept test detects unmeasured horizontal pleiotropy, and when the intercept is significant (*P* < 0.05), the result is implausible^31^. MR-PRESSON is more stringent compared to the Weighted median method and evaluates whether causality values have changed by identifying and excluding correcting outlier SNPs^32^. Therefore, diets effect on disease only when two or more statistical methods are in the same direction. If only the IVW approach yields support for the influence of a factor, and the other approaches are consistent in the direction of the beta, based on previous research, we consider this factor to be a potential influencing factor^33,34^. Cochran's Q test assessed heterogeneity in the IVW model. Cochran's Q test P < 0.05 indicates the presence of heterogeneity. The presence of heterogeneity does not negate the accuracy of the results because heterogeneity is unavoidable. All analyses were performed in R software (version 4.2.1) using the TwoSampleMR package (version 0.5.7).

### MR estimates using multivariable MR

The multivariable MR approach, serving as an extension of univariable MR, enables the assessment of shared causal effects of various risk factors on respiratory disease risk by integrating all relevant exposures into a single model. In order to explore the impact of univariable MR results in smoking and non-smoking populations and to control for possible bias from education, we employed multivariable MR analysis to estimate the direct effect of dietary factors on respiratory disease risk. We processed instrumental variables for smoking and education as follows: first, we extracted instrumental variables related to smoking and education from the whole genome and integrated them with existing instrumental variables related to respiratory diseases. Next, to ensure independence between instrumental variables, we excluded duplicate variants and aggregated according to linkage disequilibrium (r^2^ < 0.001, using estimates from the 1000 Genomes European Reference Group). To further enhance the statistical efficacy of the SNPs, we removed weak instrumental variables and selected those with F-values greater than 10 for subsequent analysis The effect estimates and corresponding standard errors for each SNP were derived from the data associated with the respective exposure and outcome. Causal effects in the multivariable MR analyses were estimated using the IVW and MR-Egger methods^35^. The MR analysis was conducted using the “TwoSampleMR (version 0.5.7)” and "MendelianRandomization" packages (version 0.8.0)^36,37^. The multivariable MR analysis provided a comprehensive assessment of the joint impact of multiple risk factors, facilitating the estimation of the direct influence of dietary factors on respiratory disease risk.

### Mediation analysis

For significant MR associations, we employed two-step MR approach to assess the extent to which the effect of dietary factors on the risk of respiratory diseases is mediated by BMI. In the first step, we screened for SNPs with significant effects from the exposure factors and excluded SNPs with knock-on imbalances. We then extracted information on the remaining SNPs in the mediator variable and calculated the causal effect of the exposure factors on the mediator variable (assuming beta1). In the second step, we looked for SNPs with significant effects from the mediator variable, again excluding SNPs with chain imbalance. We then extracted information on the remaining SNPs in the outcome variable and calculated the causal effect of the mediator variable on the outcome (assuming beta2). From previous studies, we derived the causal effect of the exposure factor on the outcome (beta0). If beta0, beta1, and beta2 are all significant, this suggests that there is a causal association between the exposure factor and the outcome, and that part of this association may be mediated by the mediating variable. This mediating effect is known as an incomplete mediation effect. If beta0 is not significant, but both beta1 and beta2 are significant, this suggests that the association between the exposure factor and the outcome is fully mediated by that mediating variable. This mediating effect is referred to as a complete mediating effect. If beta0 is significant, but one of beta1 and beta2 is not significant, this means that the association between the exposure factor and the outcome is not mediated by the mediating variable. By employing this comprehensive two-step MR analysis, we gained insights into the potential mediating role of BMI in the relationship between dietary factors and respiratory diseases. It facilitated our understanding of how BMI may mediate the complex interplay between dietary factors and respiratory health outcomes, shedding light on the mechanisms underlying these associations.

## Results

### Univariable MR

We used two-sample MR to analyses the effects of 20 dietary factors on the respiratory system. In this study, the number of SNPs ranged from 11 to 62, and only SNPs with F > 10 were used in the analysis. The study population consisted of individuals of European descent, with sample sizes ranging from 114,999 to 607,291 for the various exposures, and minimal overlap existed between the populations involved in the exposures and the outcome, which was sourced from the FinnGen biobank.

We conducted detailed analyses to identify dietary factors that may promote or exacerbate COPD. Notably, dried fruit intake demonstrated a protective effect on COPD (OR: 0.211; 95% CI 0.117–0.378; *P* < 0.001), a finding corroborated by the Weighted median method (OR: 0.196; 95% CI 0.096–0.399; *P* < 0.001). However, the MR-Egger method yielded insignificant results in this context. Conversely, alcohol intake frequency was associated with an increased risk of COPD exacerbation (OR: 1.283; 95% CI 1.023–1.067;* P* < 0.035), as confirmed by the IVW method (other methods were consistent in the direction of the beta). In conclusion, dried fruit intake is a potential protective factor for COPD, while alcohol intake may increase the risk of COPD. Further details can be found in Table 1 and Supplementary Table S3.

**Table 1:** Causal effects of dietary factors on COPD via univariable MR analyses.

Phenotype	IVs	OR (95%CI)	Beta (SE)	*P*	Q test
Coffee intake					
IVW	38	1.110 (0.704–1.750)	0.232	0.653	0.282
Weighted median	38	0.994 (0.157–1.911)	0.333	0.986	
MR-Egger	38	0.813 (0.324–2.043)	0.467	0.663	
MR-PRESSO	38			0.656	
Global test				0.317	
Egger-intercept				0.451	
Alcohol intake					
IVW	95	1.283 (1.023–1.067)	0.115	0.035	0.001
Weighted median	95	1.201 (0.893–1.165)	0.151	0.224	
MR-Egger	95	1.592 (0.787–3.128)	0.395	0.198	
MR-PRESSO	95		0.248	0.033	
Global test				0.033	
Egger-intercept				0.526	
Tea intake					
IVW	40	0.910 (0.607–1.362)	− 0.095	0.646	0.088
Weighted median	40	0.694 (0.404–1.192)	− 0.366	0.186	
MR-Egger	40	0.812 (0.331–1.992)	− 0.208	0.652	
MR-PRESSO	40		− 0.806	0.648	
Global test				0.090	
Egger-intercept				0.782	
Dried fruit					
IVW	41	0.211 (0.117–0.378)	0.298	1.78E − 07	0.115
Weighted median	41	0.196 (0.096–0.399)	0.364	7.37E − 06	
MR-Egger	41	0.344 (0.024–4.899)	1.356	4.36E − 01	
MR-PRESSO	41		− 1.557	5.83E − 06	
Global test				0.1337	
Egger-intercept				0.714	

*CI* confidence intervals, *IVs* instrumental variables, *IVW* inverse-variance weighted, *MR* mendelian randomization, *MR-PRESSO* Pleiotropy Residual Sum and Outlier, *OR* odds ratio, *SE* standard error, *SNP* single-nucleotide polymorphism.

Beta is the estimated effect size. P < 0.05 were considered statistically significant.

Currently relevant studies have not demonstrated the pathogenesis of IPF, therefore, we used MR to analyze the effects of 20 dietary factors on IPF. Interestingly, we found that pork intake could promote IPF, which was jointly confirmed by IVW (OR: 1.051*10^2^, 95% CI 4.354–2.536*10^3^; *P* = 0.004) and Weighted median (OR: 1.345*10^2^; 95% CI 2.443–7.404*10^3^; *P* = 0.016). However, other dietary factors did not show a correlation with the development of IPF. Supplementary TableS4 contains further information.

**Figure 2:**
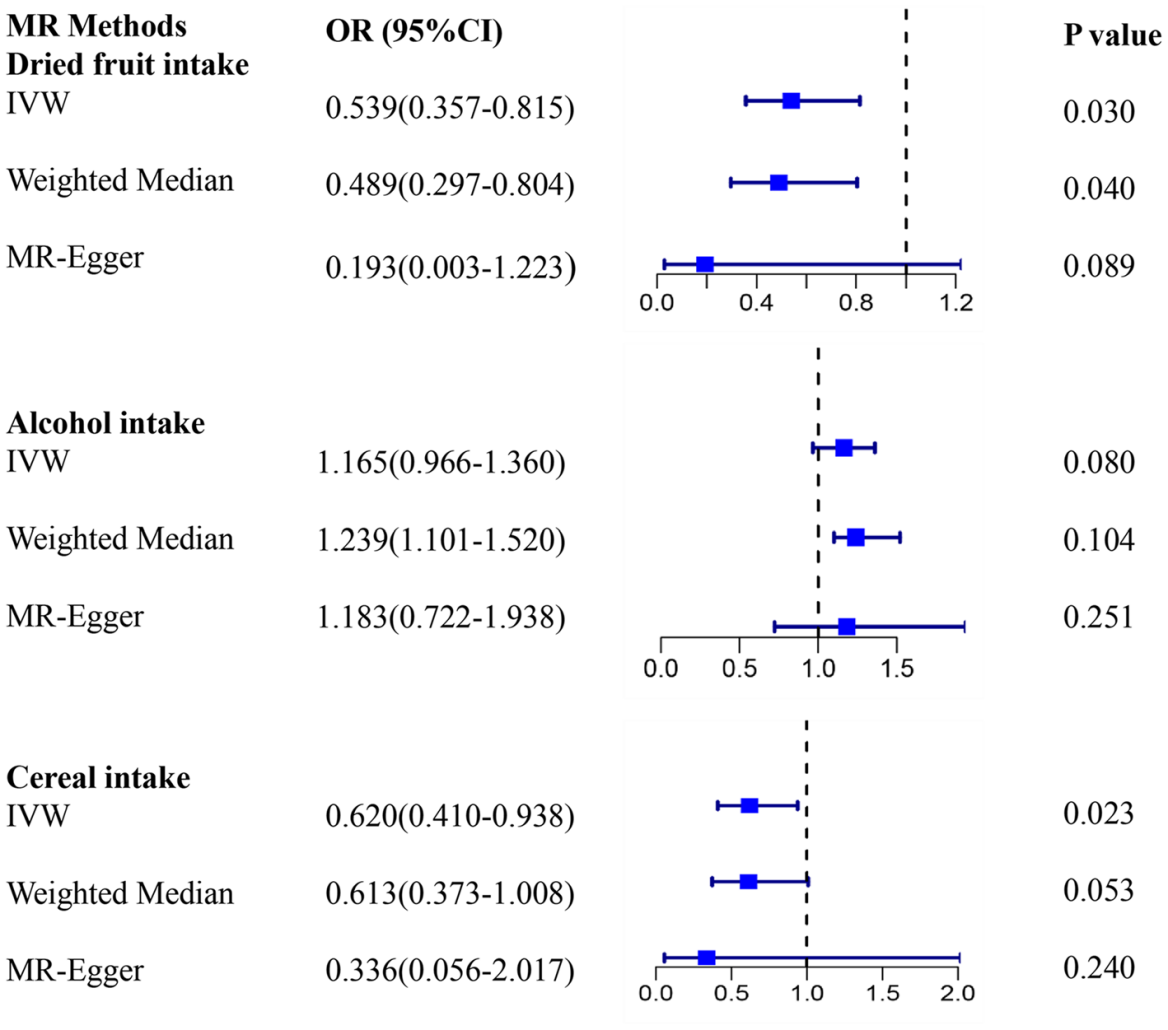
Plot of MR analyses for dietary that were associated with asthma risk by IVW Weighted median and MR-Egger.

Similarly, we used MR to analyze the effects of 20 dietary factors on asthma, and unlike previously known risk factors, we identified other factors that may influence asthma. Specifically, we discovered that eating dried fruit could help prevent the development of asthma (Fig. 2 and Supplementary Table S5). Furthermore, we screened for several other factors that may influence the development of asthma. For example, we observed cereal intake may alleviate asthma attacks to a certain extent, while alcohol intake may be associated with an increased incidence of asthma (Fig. 2). However, due to the limitations of the current statistical methods, we only observed the relationship between these associations between diets factors and asthma on a particular statistical method (The IVW method provides support, with the beta values from the other methods consistently aligning in the same direction.). Therefore, there is a need to further investigate the role of these three dietary factors in determining their influence on asthma occurrence.

Unfortunately, as stated in the previous article, at least two statistically significant approaches were required to consider dietary factors to be connected with OSA, and we did not discover that a specific dietary component may influence the development of OSA. However, we found several dietary factors that exhibited potential associations with the remission of OSA. Specifically, cheese intake and dried fruit intake were among the factors that showed possible links to the remission of OAS. For detailed information, please refer to Supplementary Table S6. Although these dietary factors were only supported by the IVW method, the rest of the methods were consistent in the direction of the beta values. Therefore, we still believe that these diets may be associated with the occurrence of OSA and need to be comfirmed by futher studies.

### Multivariable MR

Because of the significant impact of smoking on respiratory disorders, we added smoking as one of the confounders to see if the previous findings differed between smoking and non-smoking groups. At the same time, several recent studies have suggested that education has a role in the development of various diseases, thus we added education as one of the confounding factors to adjust for the influence of education. After smoking was eliminated, the effects of dried fruit consumption on COPD (IVW OR: 0.276; 95% CI 0.141–0.537; *P* < 0.001) and asthma (IVW OR: 0.531; 95% CI 0.348–0.811; *P* < 0.001) remained. Similarly, the impact of pork intake on IPF remained significant (IVW OR: 5.215; 95% CI 2.698–7.731; P < 0.001) even after controlling for smoking, suggesting that pork intake is associated with an increased risk of IPF progression. Interestingly, after adjusting for education, the effects of dried fruit intake on COPD (IVW OR: 0.180; 95% CI 0.096–0.340; *P* < 0.001) and pork intake on IPF remained (IVW OR: 0.631; 95%CI: 0.355–1.122; *P* < 0.001). However, the effect of dried fruit intake on asthma did not remain significant (IVW OR: 0.631; 95% CI 0.355–1.122; *P* = 0.117). Furthermore, when we controlled for both smoking and education, the effects of dried fruit intake on COPD (IVW OR: 0.185; 95% CI 0.090–0.378; *P* = 0.001) and pork intake on IPF persisted (IVW OR: 5.026; 95% CI 2.544–7.508; *P* = 0.001). However, the association between dried fruit intake and asthma was not significant (IVW OR: 0.565; 95% CI 0.339–0.943; *P* = 0.994) (Table 2, Supplementary Tables S7, S8). These results highlight the importance of considering educational factors in assessing the relationship between dietary factors and respiratory diseases, and they provide further insights into the varying impacts of specific dietary components on different respiratory conditions. The results of MR-Egger were similar to IVW, which further argues for the accuracy of our results.

**Table 2:** Causal effects of education and smoking on vascular dementia via multivariable IVW MR analyses.

MVMR	Disease	SNPs	MVMR-IVW OR (95%CI)	*P*	MVMR-Egger OR (95%CI)	*P*	Intercept
MVMR1	COPD	106	0.276 (0.141–0.537)	0.001	0.261 (0.133–0.511)	0.001	0.162
	IPF	92	5.215 (2.698–7.731)	0.001	4.973 (2.435–7.521)	0.001	0.219
	Asthma	106	0.531 (0.348–0.811)	0.003	0.522 (0.339–0.799)	0.003	0.679
MVMR2	COPD	52	0.180 (0.096–0.340)	0.001	0.288 (0.098–0.848)	0.024	0.294
	IPF	32	4.514 (1.898–7.130)	0.001	4.374 (1.723–7.026)	0.001	0.529
	Asthma	52	0.631 (0.355–1.122)	0.117	-0.832 (0.311–2.223)	0.715	0.496
MVMR3	COPD	112	0.185 (0.090–0.378)	0.001	0.192 (0.094–0.392)	0.001	0.240
	IPF	100	5.026 (2.544–7.508)	0.001	4.837 (2.377–7.370)	0.001	0.294
	Asthma	112	0.565 (0.339–0.943)	0.994	0.565 (0.342–0.934)	0.026	0.921

*MVMR 1* multivariable MR adjusting for smoking, *MVMR 2* multivariable MR adjusting for education, *MVMR 3* multivariable MR adjusting for smoking and education.

### Median analysis

**Figure 3:**
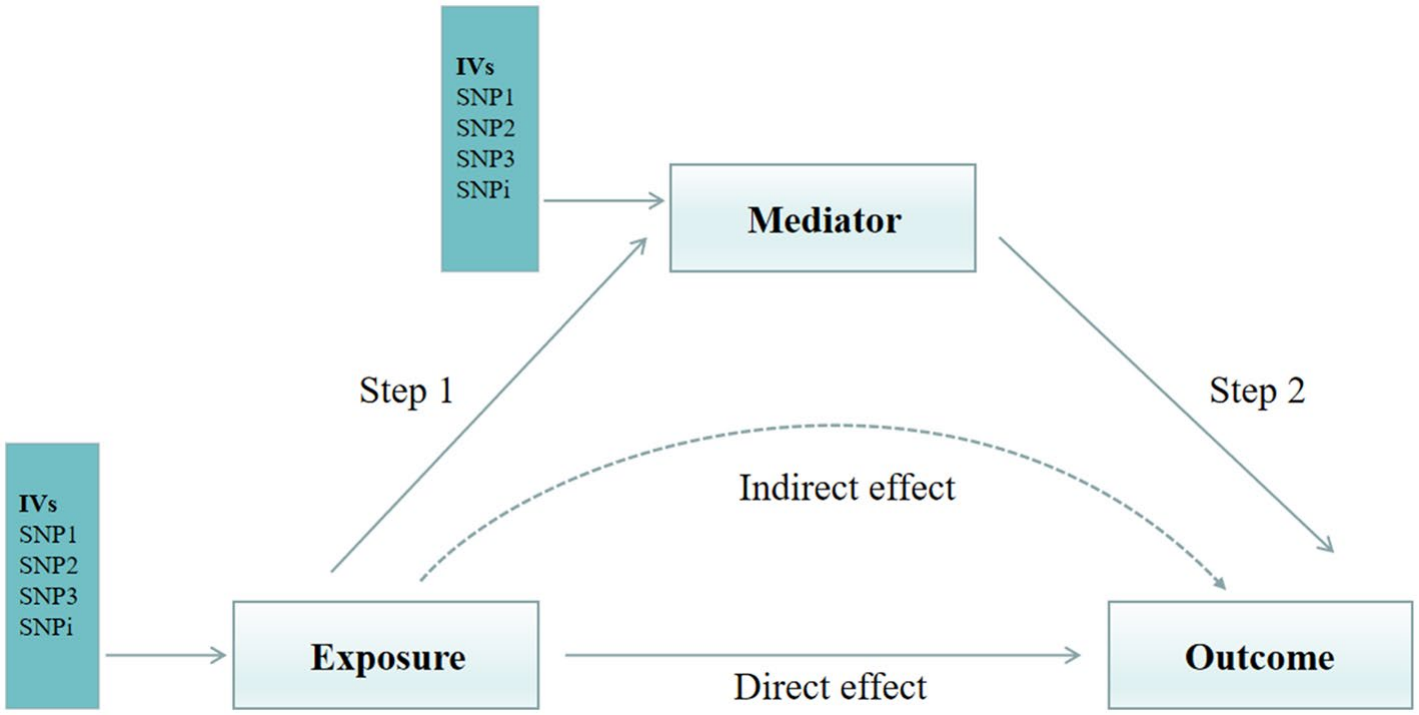
The design of mediation analysis of dietary factors on respiratory diseases.

Since different diets have a significant effect on BMI, mediation analyses were conducted to further explore whether BMI could be used as a mediating variable to influence the results (Fig. 3). Specifically, dried fruits were able to significantly influence BMI (OR: 0.675; 95% CI 0.469–0.918; *P* = 0.012), while BMI was able to influence the occurrence of COPD (OR: 1.812; 95% CI 1.596–2.056; *P* < 0.001). Therefore, BMI could serve as a mediating variable for the effect of dried fruit on COPD. Similarly, we did the same analyses in asthma and IPF, and the results showed that BMI could serve as a mediating variable for dried fruit to influence the occurrence of asthma, but not as a mediating variable for pork intake to influence IPF. In addition, we performed MR of blood glucose to explore the effect of dietary factors on blood glucose and the mediating role of blood glucose in respiratory diseases. However, the results of the study did not find a mediating role for blood glucose in respiratory diseases. There are still many uncharted areas of mediating variables for the effect of dietary on respiratory diseases that need to be explored in further future studies. Specific data results can be found in Supplementary Tables S9, S10.

## Discussion

This large-scale MR study delves into the potential causal role of dietary factors in a wide range of respiratory diseases. We have identified several dietary factors that may impact susceptibility to respiratory system diseases. Notably, dried fruit intake exhibited a protective association against the development of COPD and asthma, while pork intake was associated with the progression of IPF. However, no significant dietary factors were found to play a role in OSA. Subsequently, we rigorously controlled for education and smoking to exclude their potential confounding effects. After correcting for educational factors, the protective effect of dried fruit intake on asthma was observed to diminish, suggesting an influence of education in this relationship. Furthermore, we investigated BMI as a mediating variable. We found that BMI could serve as an incomplete mediator for the effects of dried fruit intake on COPD and asthma while not acting as a mediator for the impact of pork intake on IPF.

We found a protective association between dried fruit intake and COPD predicted at the genetic level. These findings align with the work of Xin et al., who also observed a link between higher fruit intake (including dried fruit intake) and a reduced likelihood of developing COPD^38^. Moreover, prospective investigations reinforced this notion, emphasizing fruit consumption as a safeguard against COPD^39^. Notably, the results of both prospective and cross-sectional studies suggest that smoking is one of the important risk factors promoting the development of COPD, so we performed multivariate MR analyses to explore whether the relationship was altered in the smoking versus non-smoking population^40^. Encouragingly, the negative association between high fruit intake and genetic prediction remained significant in the multivariate model, suggesting that fruit may be one of the independent protective factors for COPD. Furthermore, previous research has posited that the heightened presence of antioxidants in fruits may mitigate the inflammatory response by reducing basal levels of oxidative stress^41,42^. This mechanism could partially explain why fruits exhibit this protective role against COPD. Despite the compelling evidence presented in our study, further research endeavors are warranted to elucidate the intricate molecular mechanisms underpinning this phenomenon.

The diagnosis of IPF, a chronic progressive fibrotic lung disease, requires careful exclusion of secondary causes of pulmonary fibrosis^43^. A large number of previous studies have demonstrated that gastro-esophageal reflux may be associated with the development of IPF^44^. However, to the best of our knowledge, the role of dietary factors on IPF has not yet been reported. Using MR analysis, we found that pork intake could promote the development of IPF. In the Asia–Pacific region, the estimated incidence of IPF per 10,000 individuals ranges from 0.35 to 1.30, whereas in Europe this figure was only 0.09–0.49^45^. This discrepancy may be partly attributed to the distinct dietary and cultural preferences between the Asia–Pacific and European regions. The Asia–Pacific population is more favorable to pork intake, while Europeans are dominated by beef and lamb. The epidemiological findings are consistent with our findings that pork intake may contribute to the development of IPF to some extent. Therefore, we believe that pork intake may increase the risk of IPF.

Asthma is a chronic respiratory disease characterized by airway inflammation and hyperresponsiveness^46^. Observational studies have provided evidence suggesting that the consumption of vegetable-based foods may delay the onset of asthma^47^. However, the results of MR did not occur that the consumption of raw vegetables intake slowed the onset of asthma. We posit that several factors may account for the difference between MR and observational studies regarding the relationship between raw vegetable intake and asthma: 1. Observational studies are confounded by a multitude of confounders; 2. MR results analyzed a wide range of vegetable intake, and observational studies examined one vegetable-based food alone, which is something that MR analyses are unable to realized. Therefore, more research evidence is needed to justify this in the future. Similar to the results of previous MR analyses, our study also demonstrated that the intake of dried fruits can slow down the onset of asthma to a certain extent^48^. Unfortunately, through MR analysis, we did not find an exact dietary factor associated with the onset of OSA, and only explored some factors that may influence it. Therefore, further research could be conducted in this direction in the future to see if dietary therapy can actually influence the progression of OSA.

Our study benefits from several strengths, notably the MR design, which minimizes residual confounding and reverse causation in observational studies. Including data from diverse sources further strengthens the reliability and robustness of our results, while limiting the population to European individuals helps mitigate ethnic heterogeneity. Additionally, we performed multivariate MR analyses to account for the potential influence of education on dietary preferences in smokers and non-smokers. However, our study has limitations, including the inability to further disaggregate dietary components and explore potential gender-specific effects and the need for further research to unravel underlying mechanisms. Our findings provide valuable insights into the complex interplay between dietary factors and respiratory diseases, paving the way for potential preventive and therapeutic strategies in this context.

## Conclusion

In conclusion, our comprehensive MR study provides evidence supporting the potential protective role of dried fruit intake against the risk of developing COPD and asthma. On the other hand, our findings suggest that pork intake may contribute to the progression of IPF. In addition, the effect of dried fruit intake on COPD was unchanged after adjusting for smoking and education diet, the effect of pork intake on respiratory system was unchanged. BMI was a mediating variable in dried fruit on COPD and asthma, whereas it did not play a mediating role in the effect of pork intake on IPF. In conclusion, our study reveals the role of dietary factors in respiratory diseases, which can provide a basis for subsequent dietary treatment.

### Supplementary Information

Supplementary Information.

Supplementary Tables.

## Data Availability

The GWAS data for this article are available under public license. The data source is the IEU open GWAS project (https://gwas.mrcieu.ac.uk/).
