# Author Correction: Mendelian randomization study reveals the relationship between dietary factors and respiratory diseases

**DOI:** 10.1038/s41598-024-53866-8

**Published:** 2024-02-19

**Authors:** Wei Lai, Guorui Li, Dunyu Peng, Ning Li, Wei Wang

**Affiliations:** 1 Department of Thoracic Surgery, Renmin Hospital of Wuhan University, Wuhan, 430060 China; 2 Department of Anesthesiology, Renmin Hospital of Wuhan University, Wuhan, 430060 China

Correction to: *Scientific Reports* 10.1038/s41598-023-50055-x, published online 18 December 2023

The original version of this Article contained an error in the Abstract.

“Conversely, pork intake was associated with an increased risk of international pharmaceutical federation (IPF) (OR: 1.051*10^2^, 95% CI 4.354–2.56*10^3^, *P* = 0.004).”

now reads:

“Conversely, pork intake was associated with an increased risk of idiopathic pulmonary fibrosis (IPF) (OR: 1.051*10^2^, 95% CI 4.354–2.56*10^3^, *P* = 0.004).”

The original Article has been corrected.

